# Health system productivity in sub-Saharan Africa: tuberculosis control in high burden countries

**DOI:** 10.1186/s12962-023-00485-1

**Published:** 2023-11-25

**Authors:** Esso-Hanam Atake

**Affiliations:** 1 Department of Economics Sciences, University of Lome (Togo), Lomé, Togo; 2 University of Lomé (FASEG), 01BP1515 Lomé, Togo

**Keywords:** Sub-Saharan Africa, Tuberculosis, Productivity, Hicks-Moorsteen index, Technological change, Efficiency

## Abstract

**Background:**

Sixteen of the 30 countries with a high tuberculosis (TB) burden are in Sub-Saharan Africa (SSA). Over 25% of TB deaths occur in the Africa region. This study aims to estimate the productivity changes of TB programs in 16 SSA countries where TB is endemic.

**Methods:**

We used Hicks-Moorsteen index to compute and decompose Total factor productivity (TFP), and the β-convergence and σ-convergence tests to check for convergence patterns among SSA countries.

**Results:**

We found that technological change has been the main driver of the TFP growth, and that increasing technical efficiency may be the first objective in efforts to improve TFP of TB programs. Moreover, the convergence tests reveal significant homogeneity in terms of TFP change between SSA countries studied.

**Conclusion:**

The findings suggest that improving technical efficiency of TB programs mainly calls for better resource allocation, capacity building in governance and management of programs, improved training of the health providers and stronger prevention policies. Policymakers must design models for integration of TB treatment under the universal health insurance schemes.

## Highlights

- Sixteen of the 30 countries with a high tuberculosis (TB) burden are in Sub-Saharan Africa (SSA). The main objective of this study is to estimate the productivity change of TB programs in Sub-Saharan Africa (SSA).
- We used Hicks-Moorsteen index to compute total factor productivity. We also provide findings on productivity changes in terms of convergence.
- Increasing technical efficiency of TB programs is the first objective in improving overall factor productivity. An efficient use of funds will arguably reduce the number of tuberculosis cases and TB burden.
- Action for Strengthening Good Governance of TB programs in SSA is imperative.
- Policymakers must design models for integration of TB services under the universal health insurance schemes.

## Introduction

Reforms for improving the efficiency of health systems comes from the X-Efficiency theory [1]. “X-efficiency theory is concerned with under-utilization of resources” [2]. “Measuring health care productivity is important as health is a large sector of the economy and with the majority of funding coming from public sources, the outlook for productivity growth is a critical factor in the debate about fiscal sustainability” [1]. The assumption is that greater efficiency gains in health production can be derived from dramatic organizational changes than from tinkering with reallocations of existing health inputs, even though the latter should not be neglected. While there is abundant literature on the analysis of productivity of health facilities [3–5], few studies have dealt with the productivity of health programs, such as the global tuberculosis (TB) Program. The Global TB Program aims to advance universal access to TB prevention, care and control, guide the global response to threats, and promote innovation.

TB is one of the top ten causes of death in the world [6]. In 2016, an estimated 10.4 million people worldwide contracted the disease, and that 1.7 million of them died [7]. Low and middle-income countries were the major victims. Over 95% of TB deaths occur in low and middle-income countries [7]. Sub-Saharan Africa (SSA) is among the regions that are highly vulnerable to TB. In 2016, the incidence of TB in this region was estimated at 25%, far higher than in Europe (3%) and America (3%). It is home to 74% of all HIV-positive TB patients reported worldwide in 2014 [6]. 21 out of 30 high TB/HIV burden countries are SSA countries [7].

World Health Organization's (WHO) universal strategy to combat TB and the Sustainable Development Goals (SDGs) advocate the eradication of TB over the period 2016–2035. The specific targets are to reduce TB deaths by 90% and the incidence of TB by 80% by 2030 [6]. This global fight calls for significant mobilization of financial resources. In 2017, funding reached US$6.9 billion in 118 low and middle-income countries, which is more than double the resources available in 2006 [6].

Financial resource mobilization is supported by the fact that the TB strategies implemented are cost-effective [8, 9]. Drug-susceptibility testing (DST) methods generate substantial cost savings in settings of high prevalence of multidrug-resistant tuberculosis [8]. Directly Observed Therapy Shortcourse (DOTS) treatment of cases with negative and extra-pulmonary bacilloscopy, and the DOTS-Plus treatment of multidrug-resistant (MDR) cases have been shown to be cost-effective in developing countries [10, 11]. Furthermore, studies have shown that the administration of bedaquiline to all patients with Multidrug—and rifampicin-resistant tuberculosis (MDR/RR-TB) could increase the success rate [12]. Microscopic Observation Drug Susceptibility (MODS) and Xpert MTB/RIF (Xpert) for detection of Mycobacterium tuberculosis and rifampicin resistance are cost-effective for diagnosis of pulmonary tuberculosis among HIV patients [13]. Consequently, despite the significant mobilization of financial resources recorded, it is acknowledged that, in order to achieve the SDG targets, substantial additional investments are needed to step up case finding and implement all these interventions on a larger scale [10].

Looking at the TB indicators of SSA as opposed to other regions of the world, one important question comes to mind: would a substantial increase in the funds for TB programs suffice to reduce significantly TB death in SSA by 2030? This question arises in the light of the evidence that health systems and programs in SSA have to contend with weak financial management, inefficient use of resources and inadequate coordination mechanisms to coordinate partner support [14, 15]. Corruption plays a major role in health-care systems in Africa [14]. There is also a lack of organization and of effective management of health services, which coupled with the above-mentioned weaknesses, has led to a situation where 47% of the population have not access to quality health services [14–16]. There are also other important factors such as low levels of awareness about the symptoms, transmission and prevention of tuberculosis and low levels of community involvement [17, 18]. TB programs will require not just more money for prevention and treatment but more value for money. 

This paper attempts to evaluate the productivity of TB programs in SSA. For the TB programs that produces several outputs using several inputs, Total factor productivity (TFP) can be measured as the ratio of an aggregate output to an aggregate input. Furthermore, we used Beta convergence and sigma convergence tests to examine the convergence patterns of productivity change of TB programs in SSA [19].

## Methods

### The Hicks-Moorsteen total factor productivity change (HMTFPC) index

Measurement of productivity change of a health program involves a comparison of the amount of outputs produced by the program and the amount of inputs used to produce those outputs over time.

Suppose we analyze the productivity change over two periods represented by $t$ and $t + 1$. For period $t$ we define an input vector as $x^t \in R_+^m$ and an output vector as $y^t \in R_+^s$.

We assume that, for each period, we observed $n$ countries with different inputs and outputs represented in period $t$ as $(x_j^t ,y_j^t )$ which comes from the reference technology $T^t = \left\{ {(x^t ,y^t ) \in R_+^m \times R_+^s :x^t produces\left. {y^t } \right\}} \right.$.

In particular, $T^t$ is estimated in Data Envelopment Analysis (DEA) as $T^t = \left\{ {(x^t ,y^t ) \in R_+^m \times R_+^s :\sum_{j = 1}^n {\lambda_j x_j^t \le x^t ,\sum_{j = 1}^n {\lambda_j y_j^t \ge y^t ,\sum_{j = 1}^n {\lambda_j = 1,\left. {\lambda_j \ge 0} \right\}} } } } \right.$ under the assumption of variable returns to scale (VRS) [20] and as $T^t = \left\{ {(x^t ,y^t ) \in R_+^m \times R_+^s :\sum_{j = 1}^n {\lambda_j x_j^t \le x^t ,\sum_{j = 1}^n {\lambda_j y_j^t \ge y^t ,\left. {\lambda_j \ge 0} \right\}} } } \right.$ under the assumption of constant returns to scale (CRS) [21].

The productivity index most commonly used in the literature is the Malmquist index [22, 23]. The Malmquist index introduced by Caves et al. [24] was popularized by Fare et al. [25] by providing a decomposition of this index into changes in technological progress and technical efficiency. In its definition, this index is based on the distance function defined by Shephard [26] to represent a technology whose most popular forms are input or output oriented.

The Malmquist output-oriented productivity index [24] is defined as follows:

1 $$M_I^{t,t + 1} (x^t ,y^t ,x^{t + 1} ,y^{t + 1} ) = \left[ {\frac{D_I^t (x^t ,y^t )}{{D_I^t (x^{t + 1} ,y^{t + 1} )}}\frac{{D_I^{t + 1} (x^t ,y^t )}}{{D_I^{t + 1} (x^{t + 1} ,y^{t + 1} )}}} \right],$$

where, $D_I^k (x^h ,y^h ) = \sup \left\{ {\tau :(\frac{x^h }{\tau },y^h ) \in T^k } \right\}$ is Shepard's input-oriented distance function calculated from the point $(x^h ,y^h ),h = t,t + 1$ at the frontier of the technology with time $k,k = t,t + 1$.

The decomposition of (1) into an efficiency change component and a technological change component is given by:

2 $$M_I^{t,t + 1} (x^t ,y^t ,x^{t + 1} ,y^{t + 1} ) = \frac{{D_I^t (x^t ,y^t )}}{{D_I^{t + 1} (x^{t + 1} ,y^{t + 1} )}}\left[ {\frac{{D_I^{t + 1} (x^{t + 1} ,y^{t + 1} )}}{{D_I^t (x^{t + 1} ,y^{t + 1} )}}\frac{{D_I^{t + 1} }}{{D_I^t (x^t ,y^t )}}} \right]^{1/2} ,$$

A value of $M_I^{t,t + 1} (x^t ,y^t ,x^{t + 1} ,y^{t + 1} ) > 1$ indicates an increase in productivity over period *t* to period *t* + 1, $M_I^{t,t + 1} (x^t ,y^t ,x^{t + 1} ,y^{t + 1} ) \prec 1$ a decrease and $M_I^{t,t + 1} (x^t ,y^t ,x^{t + 1} ,y^{t + 1} ) = 1$ an unchanged level of productivity.

“Although the Malmquist index can be interpreted as a measure of productivity change over time, it should not be regarded as a total factor productivity (TFP) measure. In a multidimensional context, TFP is usually defined as the ratio of an aggregate output to an aggregate input. This definition naturally leads to TFP indices that can be expressed in terms of the ratio of an output quantity index over an input quantity index” [22]. The Malmquist index is not an adequate total factor productivity (TFP) measure [27, 28]. The Malmquist index generally suffers from several infeasibilities during its application, which are due to its input and output distance functions that could sometimes be undefined. Moreover, “the Malmquist index and its technological change component are also criticized for not fulfilling the determinateness axiom. This drawback is related to the fact that the Shephard distance functions can yield infeasible results when mix periods are evaluated [22]. These problems have been reported in the literature [28, 29].

To solve the problem of infeasibility and interpretation often encountered when using Malmquist indices as a TFP index, Bjurek [29] proposed the *Hicks-Moorsteen Productivity (HMTFPC)* index [30]. The Hicks-Moorsteen Total Factor Productivity (*HMTFPC*) index was introduced with the aim of overcoming all the above weaknesses of the traditional Malmquist index. The HMTFPC index is measured as follows:

3 $$HM^{t,t + 1} (x^t ,y^t ,x^{t + 1} ,y^{t + 1} ) = \frac{{QI^{t,t + 1} (x^t ,y^t ,x^{t + 1} ,y^{t + 1} )}}{{XI^{t,t + 1} (x^t ,y^t ,x^{t + 1} ,y^{t + 1} )}} = \frac{{\left[ {\frac{{D_o^I (x^t ,y^{t + 1} )}}{D_o^I (x^t ,y^t )}\frac{{D_o^{t + 1} (x^{t + 1} ,y^{t + 1} )}}{{D_o^{t + 1} (x^{t + 1} ,y^t )}}} \right]^{1/2} }}{{\left[ {\frac{{D_I^t (x^{t + 1} ,y^t )}}{D_I^t (x^t ,y^t )}\frac{{D_I^{t + 1} (x^{t + 1} ,y^{t + 1} )}}{{D_I^{t + 1} (x^t ,y^{t + 1} )}}} \right]^{1/2} }}.$$

The characteristics of HMTFPC index generally resolve the limitations of the traditional Malmquist index [22]. First, it can be trivially interpreted as a change in TFP, i.e., the ratio of an aggregate output change index $QI^{t,t + 1} (x^t ,y^t ,x^{t + 1} ,y^{t + 1} )$ to an aggregate input change index $XI^{t,t + 1} (x^t ,y^t ,x^{t + 1} ,y^{t + 1} )$. Second, this index satisfies deterministic properties under mild conditions [31] since, for all the input distance functions included in (3), it holds that the time period of the reference technology matches the time period of the fixed output quantity and, for all the output distance functions, the period of the reference technology is equal to the period of the fixed input quantity. Third, the HMTFPC index is well-defined even with variable returns to scale [22, 31].

Defining the HMTFPC index has another important advantage in that it is possible to determine the measures of output change, $QI^{t,t + 1} (x^t ,y^t ,x^{t + 1} ,y^{t + 1} )$, and of input change, $XI^{t,t + 1} (x^t ,y^t ,x^{t + 1} ,y^{t + 1} )$, which can be useful for a DMU, particularly in the health sector.

A value of $QI^{t,t + 1} (x^t ,y^t ,x^{t + 1} ,y^{t + 1} ) \succ 1$ indicates an increase in outputs from period *t* to period $t + 1$, and $QI^{t,t + 1} (x^t ,y^t ,x^{t + 1} ,y^{t + 1} ) \prec 1$ a decrease. With regard to the change in inputs, the values associated with $XI^{t,t + 1} (x^t ,y^t ,x^{t + 1} ,y^{t + 1} )$ are interpreted in the same way.

For the HMTFPC index, a value of $HM^{t,t + 1} (x^t ,y^t ,x^{t + 1} ,y^{t + 1} ) \succ 1$ indicates an increase in TFP, while a value less than a unit indicates a decrease in TFP.

In addition, the HMTFPC index offers several possibilities in terms of its decomposition [32]. O'Donnell [33] recently introduced a general decomposition of the HMTFPC index, which is valid for any ‘multiplicatively complete index’ [22]:

4 $$HM^{t,t + 1} (x^t ,y^t ,x^{t + 1} ,y^{t + 1} ) = \left( {\frac{{TFP^{t + 1*} }}{{TFP^{t*} }}} \right).\left( {\frac{{TFPE^{t + 1} }}{TFPE^t }} \right)$$

where, $TFP^{k*} = \max \left\{ {\left[ {\frac{{D_o^t (x^t y).D_o^{t + 1} (x^{t + 1} y)}}{{D_I^t (xy^t ).D_I^{t + 1} (xy^{t + 1} )}}} \right]} \right.^{1/2} :(x,y)\left. { \in T^k } \right\}$, $k = t,t + 1$ represents TFP at the point of maximum productivity in the period $k$, and $TFPE* = \frac{{\left[ {\frac{{D_o^t (x^t .y^k ).D_o^{t + 1} (x^{t + 1} .y^k )}}{{D_I^t (x^k .y^t ).D_I^{t + 1} (x^k .y^{t + 1} )}}} \right]^{1/2} }}{{TFP^{k*} }}$, is the so-called TFP efficiency and generally represents the measure of DMU performance.

The first component in brackets to the right of Eq. (4) is interpreted as a measure of the change in the maximum TFP over time, which represents the natural measure of technological change [22]. The second component may be interpreted as a measure of overall efficiency change [33].

In this paper, we use O'Donnel [33] decomposition which is implemented in our empirical analysis using DPIN 3.0 software. Finally, following Aparicio et al. [22] who defined a base period HMTFPC index by fixing a baseline period for technology.

### Convergence test

In this section, we present the tests used to analyze the convergence over time of the productivity change index across countries studied. We use the $\beta { - }convergence$ and $\sigma { - }convergence$ tests proposed by Barro and Sala-i-Martin [34, 35].

The $\beta { - }convergence$ establishes a relation between the productivity change index growth rate with respect to the initial period of productivity change. The objective is to verify whether the productivity change index for countries with lower levels of productivity change in the first period grows at a faster rate than for countries with the best initial productivity change index scores [22]. If the $\beta$ coefficient is negative and statistically significant, convergence is established, otherwise it is a divergence. The regression function [22] used to compute $\beta { - }convergence$ when analyzing productivity change with the Hicks-Moorsteen index is defined as follows:

5 $$\ln HM_{i,t} - \ln HM_{i,t - 1} = \alpha + \beta \ln HM_{i,t - 1} + \varepsilon_{i,t}$$

where $\ln HM_{i,t}$ is the logarithm of the Hicks-Moorsteen TFPC index of country $i$ at period $t$; $\ln HM_{i,t - 1}$ is the logarithm of the Hicks-Moorsteen TFPC index of country $i$ at period $t - 1$; $\alpha$ and $\beta$ represents the factors to be estimated, and $\varepsilon_i$ the error term. By referring to Kumar and Russel [36] and Aparicio et al. [22] we used the generalized least squares (GLS) method to make calculations whenever errors were correlated and/or there was inequality in change.

As for the $\beta { - }convergence$, it represents the estimate of the cross-sectional dispersion. It indicates the speed at which a country's productivity change converges with the average productivity change of the sample [22]. The $\beta { - }convergence$ is defined as follows [28]:

6 $$\sigma_t = \sqrt {{\frac{{\sum_{i = 1}^N {(\ln HM_{i,t} - \mu_t )^2 } }}{N}}}$$

where, $N$ is the total number of countries considered in this study and $\mu_t$ is the sample mean of $\ln HM_{i,t}$. There will be $\sigma - convergence$ if standard deviation decreases over time.

### Data and variables

#### Data

The World Health Organization (WHO) has published a global TB report every year since 1997. The purpose of the report is to provide an assessment of the TB epidemic and progress in TB diagnosis, treatment and prevention efforts, as well as an overview of TB-specific financing and research. It also discusses the broader agenda of universal health coverage, social protection and other SDGs that have an impact on health. Data were available for 202 countries and territories that account for over 99% of the world’s population and TB cases. Our paper covers the period 2009 to 2016, i.e. 8 years—data required for this paper are available over this period and comes from the annual reports of global TB Program. This study covers 16 SSA countries where TB is endemic: Angola, Democratic Republic of Congo, Ethiopia, Kenya, Mozambique, Nigeria, South Africa, United Republic of Tanzania, Central African Republic, Congo, Lesotho, Liberia, Namibia, Sierra Leone, Zambia and Zimbabwe.

### Variables

Given the dearth of studies on the productivity of anti-TB programs, the choice of variables comes only from World Health Organization (WHO) literature. Considering that TB-related deaths among HIV-positive people are officially classified as HIV/AIDS-related deaths in the international classification of diseases, this study focuses only on HIV-negative TB.

### Choice of inputs

Three categories of factors could be considered as inputs for TB programs: financing TB control, diagnosis and treatment. Financing is a composite of domestic and international funding. In SSA, funding is mainly from the Global Fund. SSA countries where TB is endemic operate under severe financial constraints and have to compete with other health programs for budget allocations from the government and donors.

Regarding diagnosis and treatment of TB, care of patients with tuberculosis (TB) starts with a quality assured diagnosis. Successful DOTS expansion, as well as programmatic management of drug-resistant and HIV-associated TB therefore require—at its core—a robust network of TB laboratories with adequate biosafety, modern methods for diagnosis, standard operating procedures and appropriate quality assurance. Nowadays, the WHO recommends rapid tests to determine whether individuals/patients are eligible for the appropriate treatment regimen at lower costs [37]. In some countries, TB diagnostic and follow-up tests are free or fully covered, while in others, patients incur substantial direct and indirect costs [38]. In many low-income countries, access to diagnostic services is difficult particularly in cases of MDR-TB, due to a lack of laboratories [39]. In SSA, the number of laboratories providing TB diagnostic services using smear microscopy and GeneXpert has gradually increased since 2009 [40]. Moreover, depending on the case and the severity of the disease, there are several types and levels of treatment: treatment of new smear-positive cases only under DOTS, smear-positive-plus DOTS-plus treatment, smear-positive-plus treatment of smear-negative cases under DOTS, DOTS treatment of smear-negative cases plus DOTS-plus standardized second-line drug re-treatment, etc. Because of correlations (covariances) of the variables and the lack of some data for all the countries studied, we used the following inputs: *National TB budget, TB treatment coverage, Number of drug susceptibility testing laboratories for which External Quality Assessment (EQA) was carried out, and the Number of laboratories providing tuberculosis diagnostic services using smear microscopy and GeneXpert.*

### Choice of outputs

In this study we use two indicators as outputs: *case fatality ratio (CFR) and TB treatment success rate (new TB cases)*.

There are two measures used to assess the proportion of infected individuals with fatal outcomes. The first is infection fatality ratio (IFR), which estimates this proportion of deaths among all infected individuals. The second is case fatality ratio (CFR), which estimates this proportion of deaths among identified confirmed cases. The TB CFR is defined as the proportion of TB patients dying due to TB. The CFR is a key indicator for monitoring progress made in view of the 2020 and 2025 SDG targets. A CFR of 6.5% is required to meet the global 2025 target to reduce deaths and TB cases. This indicator measures the variation of equity in access to TB diagnosis and treatment across countries because, if all TB patients had access to rapid diagnosis and high-quality treatment, the prevalence rate would be low in all countries [6]. With regard to the treatment success rate, WHO considers that high health coverage for appropriate treatment is a fundamental requirement for achieving the targets and objectives of the End TB Strategy. WHO recommends that at least 90% treatment success rate (TSR) for all persons diagnosed with TB and initiated on TB treatment services. Despite this recommendation, substantial shortfalls in TB treatment success are common.

The inputs and outputs we used are defined in the Table 1.

**Table 1:** Key statistics for inputs and outputs

Indicators	Variable name	Definition/measure	Mean	Min	Max
*Outputs*					
Case fatality ratio (%)	cfr	“Number of TB deaths divided by estimated number of incident cases in the same years, expressed as a percentage”^6^ *Recommended target level:* < = *5%* The SDG targets are to reduce cfr to 6.5% by 2025	13.07 (6.88)	3.46	30.74
TB treatment success rate (%)	tsr	“Percentage of notified TB patients who were successfully treated”^6^ *Recommended target level:* > = *90%*	78.61 (14.10)	23	91
*Inputs*					
National TB budget (US$ millions in PPP)	tbb	Public expenditure and aid from international institutions to TB Programs. All national TB budget were converted to express in a common currency the monetary aggregates. We used the Purchasing Power Parity (PPP)	31.39 (120.90)	0.43	476.04
TB treatment coverage (%)	tbc	“Number of new and relapse cases that were notified and treated, divided by the estimated number of incident TB cases in the same year, expressed as a percentage”^6^	53.00 (13.51)	22	84
TB diagnostic services	tds	Numbers of laboratories providing tuberculosis diagnostic services using smear microscopy and GeneXpert and TB diagnostic	594.17 (740.26)	17	2972
Quality	qua	Number of drug susceptibility testing laboratories for which External Quality Assessment (EQA) was carried out	2.60 (3.48)	0	16

(): Std. Dev

## Results

### Descriptive statistics

Table 1 shows an overall case fatality ratio among SSA patients with TB during treatment of 13.07% between 2009 and 2016. The treatment success rate varies between 23 and 91%. With regard to inputs, over the period studied and depending on the country, the funds allocated to TB programs varied from $0.43 to $476.04 million on average per year. The TB treatment coverage varied between 22 and 81%. The numbers of laboratories providing tuberculosis diagnostic services using smear microscopy and GeneXpert and TB diagnostic differs across countries. Moreover, some countries do not have drug susceptibility testing laboratories for which External Quality Assessment was carried out.

### Total factor productivity change (TFPC)

The HMTFPC index was calculated using 2009 as a reference base period and assuming VRS. Percentiles have been used to illustrate the distribution of the index. “This has the advantage of avoiding the biases that top- or bottom- ranking DMUs can cause with respect to mean values” [22].

Table 2 shows that there is a positive TFP index in recent years, although there was a decline in 2014. It is important to note that the largest increase in the TFP (35.7%) in 2016 with respect to the base year corresponds to the cutbacks in inputs (9.1%) and a positive change in outputs (0.9%). In general, the results reveal that during periods of decreasing TFP (8.1%, and 4.3% respectively in 2010 and 2011, with respect to the base year) there was an increase in the quantities of inputs used (5.8% and 3.6% in 2010 and 2011 respectively).

**Table 2:** Output-input changes and HMTFPC index

Component	Years	Percentiles			Mean	St.Dev
		25	Median	75		
Output Change	2009–10	0.974	0.996	1.008	0.993	0.078
	2009–11	0.979	0.999	1.011	1.027	0.128
	2009–12	0.982	0.999	1.015	0.993	0.025
	2009–13	0.983	1.000	1.023	0.978	0.07
	2009–14	0.993	1.005	1.033	1.054	0.159
	2009–15	1.000	1.008	1.036	1.082	0.096
	2009–16	1.008	1.074	1.142	1.009	0.045
Input Change	2009–10	0.908	0.983	1.004	1.058	0.133
	2009–11	0.925	1.000	1.033	1.036	0.141
	2009–12	0.951	1.002	1.056	1.022	0.138
	2009–13	0.980	1.009	1.062	1.076	0.351
	2009–14	0.984	1.016	1.079	0.982	0.095
	2009–15	0.984	1.016	1.079	1.042	0.095
	2009–16	0.987	1.024	1.081	0.909	0.283
HMTFPC	2009–10	0.664	0.995	1.1017	0.919	0.278
	2009–11	0.678	1.024	1.107	0.957	0.32
	2009–12	0.686	1.025	1.135	1.004	0.312
	2009–13	0.691	1.028	1.136	1.139	0.472
	2009–14	0.699	1.053	1.1756	0.973	0.273
	2009–15	0.765	1.074	1.247	1.026	0.267
	2009–16	0.853	1.074	1.542	1.357	1.38

### HMTFPC decomposition

An analysis of the two components of TFP, namely, technical efficiency change and technological change shows that technological change has been the main driver of the TFP growth of TB control programs in SSA (Table 3). Furthermore, over the periods 2009–2012 and 2009–2013 characterized by a decline in the TFP, the results reflect a strong improvement in the level of technical efficiency and a decline in technological change.

**Table 3:** HMTFPC index decomposition

Component	Years	Percentiles			Mean	St.Dev
		25	Median	75		
HMTFPC	2009–10	0.828	0.950	1.038	0.95	0.141
	2009–11	0.871	0.964	1.038	1.007	0.173
	2009–12	0.924	0.993	1.069	0.988	0.131
	2009–13	0.931	0.995	1.077	0.979	0.282
	2009–14	0.940	1.015	1.096	1.112	0.305
	2009–15	0.959	1.026	1.112	1.046	0.136
	2009–16	1.004	1.029	1.117	1.538	1.490
Technological change	2009–10	0.743	0.879	0.987	1.051	0.126
	2009–11	0.848	0.913	0.988	1.022	0.109
	2009–12	0.946	1.000	1.051	0.841	0.141
	2009–13	0.979	1.012	1.096	0.911	0.127
	2009–14	0.987	1.039	1.124	1.041	0.109
	2009–15	0.990	1.051	1.141	1.087	0.192
	2009–16	1.001	1.065	1.232	4.004	8.676
Efficiency change	2009–10	0.805	0.922	1.007	0.912	0.139
	2009–11	0.870	0.947	1.019	0.991	0.184
	2009–12	0.877	0.955	1.046	1.201	0.219
	2009–13	0.904	0.966	1.078	1.116	0.456
	2009–14	0.918	0.974	1.088	1.075	0.311
	2009–15	0.954	0.986	1.249	0.977	0.128
	2009–16	1.014	1.199	1.424	1.122	0.995

### Convergence tests

We carried out a convergence analysis to determine the relative position and distance of productivity change between countries at different periods.

#### β-convergence

First, we used the year 2009 as the reference base period and calculated the β-convergence at each period in relation to the base year. Next, we performed the test for each period compared to the previous one, in order to find additional evidence of convergence from one period to another [22]. The results in Table 4 show that the coefficients of β-convergence are negative and significant from one period to another. This points to a converging trend among the countries sampled, that is, the productivity change index for countries with poorer levels of productivity index in the first period grows faster than for countries with a higher initial productivity change level. Our findings therefore support the hypothesis of convergence among SSA countries in the fight against TB.

**Table 4:** $$\beta - convergence$$ coefficients

Considering 2009/10 as the baseline period						
	2009/11–2009/10	2009/12–2009/10	2009/13–2009/10	2009/14–2009/10	2009/15–2009/10	2009/16–2009/10
$\beta { - }coefficient$	0.036	− 0.101	0.046	− 0.361***	0.065	0.186

Considering the previous period instead of 2009/10						
		2009/12–2009/11	2009/13–2009/12	2009/14–2009/13	2009/15–2009/14	2009/16–2009/15
$\beta { - }coefficient$		− 0.209**	− 0.199	− 0.329***	− 0.466***	− 0.041***

***, and **: below 1%, 5% statistical significance thresholds, respectively

#### σ-convergence

Table 5 presents the 2010–2015 standard deviations of the HMTFPC index for the entire sample and the corresponding p-values for the variance ratio test with the null hypothesis that the ratio of the 2 standard deviations is equal to 1 (as opposed to the 2-sided alternative). In general, and regardless of the period, the results show a non-statistically significant difference between the standard deviations and the base year and from one period to another. Thus, it can be deduced that for the entire sample, the σ convergence occurred between 2010 and 2015.

**Table 5:** Standard deviation of SSA countries’ TFPC

Considering 2009/2010 as the baseline period			Considering the previous period instead of 2009/2010		
Standard deviation		p-value	Standard deviation		p-value
2010	0.278	0.598	2010	0.278	0.598
2011	0.320		2011	0.32	
2010	0.278	0.754	2011	0.320	0.829
2012	0.302		2012	0.302	
2010	0.278	0.064	2012	0.302	0.121
2013	0.457		2013	0.457	
2010	0.278	0.844	2013	0.457	0.042
2014	0.264		2014	0.265	
2010	0.278	0.876	2014	0.265	0.967
2015	0.267		2015	0.267	

## Discussion

Our results show that periods of strong TFP increase correspond to periods of decrease in the quantities of inputs used and increase in outputs achieved, and that periods of TFP decline correspond to increases in the quantities of inputs used. It is possible for TB programs to increase their output without any change in inputs, or decrease costs without any change in output. The findings suggest that TB programs have significant potential for gains in productivity. Additional funding would yield important outcomes through better management and better organization. Moreover, an efficient use of funds will arguably reduce the number of tuberculosis cases and eventually give a positive impact to the economy.

With financial constraints, productivity improvement need social protection measures that alleviate the financial hardships faced by many TB patients. Our results suggest that to achieve the goal of ending the epidemic once and for all, policymakers and program managers must design models for integration of TB services under the universal health insurance schemes.

Secondly, we found that technological change has been the main driver of growth in TFP over time. Digital interventions on diagnostic tools and treatment adherence technologies, such as video-observed therapy and SMS affect TB programs positively. Across Africa, affordable smartphones, digital technology, and the connective power of the internet are transforming health delivery. Available technological innovations can solve many of the programmatic and logistical barriers that have hindered TB control efforts for the past years.

Furthermore, we deduced that the threshold for increasing technical efficiency that would trigger and/or enhance the growth of TFP has not yet been reached. Overall, the results suggest that the main difficulty in raising the productivity level of TB programs lies in improving the technical efficiency level. Increasing the technical efficiency of TB programs is therefore the first objective in improving overall factor productivity. Strategies in terms of improved resource allocation, better knowledge of the production process, improved work organization and new investments to increase production capacity and technology are some of the factors likely to significantly enhance the efficiency of these programs.

With regard to resource allocation, which is still the core problem, studies have shown that in developing countries, overdiagnosis could lead to wasted resources (i.e., treatment drugs and manpower to conduct DOTS) [41]. Overdiagnosis and overtreatment due to chest x-rays could lead to an unmanageable burden on resources in poor countries such as those in SSA [36]. Studies have also established a relationship between corruption and outcomes of TB programs [42]. Corruption is considered as one of the main determinants of tuberculosis control in Asia and the Pacific [42]. In the health sector, corruption affects spending on: infrastructure construction, drug procurement, equipment, product quality regulation, services, health centers and health professionals. This negatively affects the health status of the population and the efficiency of health programs [43]. In 2020 report, the Global Fund’s Inspector General listed grave misuse of funds in four of the 145 countries which receive grants from the Global Fund; all in SSA [44]. “The Global Fund has suspended relevant grants in Mali and Zambia and terminated another grant in Mali. Special safeguards have been imposed on continuing grants in Djibouti, Mauritania and Mali, meaning that they are subject to particularly close scrutiny and restrictions on cash transfers. These safeguards are also in force in Cote d’Ivoire and Papua New Guinea” [44]. In SSA countries where health systems have to cope with weak financial management and wastage of resources [14, 15], it is important to limit corruption and manage the process of tuberculosis control in order to limit overdiagnosis and overtreatment which have serious financial consequences.

For other categories of authors, the less positive results of these programs can be attributed to poor governance [14, 17, 45]; institutional designs and organizational practices influence implementation of the national TB control program. Hence the need to strengthen the governance and management capacity of national TB control programs to ensure robust, responsive and inclusive national anti-TB systems. Governance for strengthening TB control programs in low-resource, high TB burden SSA countries is imperative. Besides, it is important to improve the training of the health providers concerned, through integrated collaborative mechanisms.

Another aspect that is just as important is TB prevention. Despite several interventions, such as the dissemination of TB messages in the media, printing and distribution of information materials, etc., populations in SSA are not sufficiently informed about the disease [17]. Removing stigma and discrimination based on TB status and improving access to TB information including through community involvement, community monitoring and social accountability for early TB diagnosis and improved treatment outcomes. The efficiency of TB programs would also depend on prevention policies aimed at strengthening communication and spreading information on the causes and symptoms of the disease, in order to control and prevent it. These awareness campaigns should target rural areas to encourage the utilization of health care services [18]. Furthermore, there is a need for more effort towards raising awareness among patients with TB about their disease while on treatment.

Finally, the convergence analysis conducted using the β-convergence and σ-convergence tests shows that countries with low productivity growth in the initial period experienced faster and more accelerated increases than the others in the sample. Moreover, these tests reveal significant homogeneity in terms of variation of the TFP.

Despite these important policy implications, this study has some limitations. First, the productivity analysis was done without taking the case mix into consideration in terms of the severity of the cases treated, the quality of care offered, the experience and qualifications of the health personnel, etc. Secondly, we were unable to obtain information on the different types of treatment in order to analyze the productivity of the different lines of treatment in a disaggregated manner. Finally, due to the unavailability of data, we were not able to disaggregate the funding allocated to national TB control programs into its specific components such as prevention, diagnosis, treatment and operating expenses, among others.

## Conclusion

The main goal of this paper was to analyze the main drivers of productivity changes of TB programs in SSA. To do this, we applied the Hicks-Moorsteen total factor productivity change index (HMTFPC). “This methodological approach is backed by good theoretical properties, but has hardly ever been used in the health context. The HMTFPC index overcomes the pitfalls of other indexes such as Malmquist index, as it is defined as a ratio of an aggregate output-quantity over an aggregate input-quantity index” [22]. This paper was carried out in 16 SSA countries where TB is highly endemic and covers the period 2009–2016. A number of results were achieved and strategies were proposed to increase the productivity of these programs. The results showed that the main difficulty in stepping up the productivity of anti-TB programs lies in improving their level of technical efficiency, and that technological change was the main source of productivity growth. Increasing the technical efficiency of TB programs is therefore the first objective in improving overall factor productivity. In this regard, it is important to implement strategies to improve resource allocation, strengthen the capacity for governance and management of national TB control programs, improve training for the health providers concerned and bolster prevention policies. Finally, governance for strengthening TB control programs in low-resource, high TB burden SSA countries is imperative.

## Data Availability

The World Health Organization (WHO) has published a global TB report every year since 1997. All data analyzed during this study comes from the annual reports of global TB Program.
